# Short Stature and Developmental Delay Associated With a Novel Frame‐Shift Mutation in ZNF292: Case Report and Literature Review

**DOI:** 10.1002/ccr3.70747

**Published:** 2025-08-07

**Authors:** Li Dongxue, Yao Ruen, Yu Ying, Chen Liting, Wang Jie, Lu Chunjiao, Li Wei, Chen Rikun, Li Cuiyun

**Affiliations:** ^1^ Department of Medical Genetics and Antenatal Diagnostic Center, Hainan Branch, Shanghai Children's Medical Center, School of Medicine Shanghai Jiao Tong University Sanya Hainan China; ^2^ Department of Medical Genetics, Shanghai Children's Medical Center Shanghai Jiao Tong University School of Medicine Shanghai China

**Keywords:** a novel de novo frameshift variant, developmental delay, short stature, whole‐exome sequencing, ZNF292

## Abstract

Pathogenic mutations in the ZNF292 gene are a significant genetic cause of Intellectual Developmental Disorder (IDD) in individuals, manifesting with a spectrum of clinical features including mild to severe intellectual impairment, speech delay, and co‐occurring autism spectrum disorder (ASD). In this study, we present a novel clinical phenotype associated with a newly identified variant of ZNF292 and conduct a thorough review of relevant literature. A 4‐year‐old female patient displayed language developmental delays, short stature, and skeletal abnormalities. Trio whole‐exome sequencing revealed a novel de novo heterozygous frameshift variant in exon 8 of the ZNF292 gene, c.5977_5978del, p.Gln1993fs. According to the ACMG guidelines, this variant is expected to be pathogenic. Our research unveils a novel variant in ZNF292‐related disorders and expands the associated phenotypic spectrum. This study highlights the significance of employing next‐generation sequencing for timely patient diagnosis, while further clinical phenotypic and genotypic investigations could improve the understanding of ZNF292‐linked conditions.

**Abbreviations**
ACMG: American College of Medical Genetics and Genomics
ASD: autism spectrum disorder
ID: intellectual disability
LOF: loss‐of‐function
NMD: nonsense‐mediated decay
P3: the third percentile value
SD: standard deviations
WES: whole‐exome sequencing

**Summary**

- This is the first case report of a novel frame shift mutation of the ZNF292 gene in Asia.
- Continuous monitoring and timely intervention for possible progressive hearing impairment is recommended, as it can produce negative effects such as behavioral problems and poor learning abilities in children's speech and language development.

## 1 Introduction

There is phenotypic and genetic heterogeneity in developmental delay and short stature, with an overlap affecting around 2%–3% of the population [1, 2, 3]. Investigations into the genetic causes of developmental delay and short stature have contributed to identifying hundreds of causative genes.

Nevertheless, there are still difficulties in making specific diagnoses due to the significant variation in clinical features. When employed as the main diagnostic tool, trio‐WES analysis can achieve a high diagnostic success rate of more than 50% in children presenting with severe developmental delays [4]. ZNF292 is one of the sequence‐specific and growth hormone‐dependent transcription factors. Research has shown a strong correlation between the silencing of ZNF292 and the presence of gain‐of‐function mutations in ZNF292 with tumorigenesis and tumor metastasis in humans [5, 6, 7]. ZNF292 encodes for a zinc finger protein that is highly conserved and functions as a transcription factor. It is prominently expressed in the developing human brain, highlighting its crucial involvement in neurodevelopment [8].

Initially, it was discovered that loss of function mutations in ZNF292 were linked to ASD in a Chinese cohort [9]. Subsequently, research and case studies validated that the ZNF292 gene is a causative gene for intellectual disability (ID) and is also connected to various other characteristics such as short stature, tone abnormalities, distinct facial features like micrognathia and hypertelorism, as well as conditions like nystagmus, esotropia, and strabismus [10, 11]. Syndromic features are heterogeneous in patients with various mutations in the *ZNF292* gene; thus, reporting novel mutations and detailed clinical information of patients could provide deep insight into this disorder.

The case reported here describes a 4‐year‐old Chinese girl who remarkably displayed short stature, language developmental delay, and skeletal anomaly due to a de novo frame shift mutation in the ZNF292 gene.

## 2 Case Presentation

**FIGURE 1:**
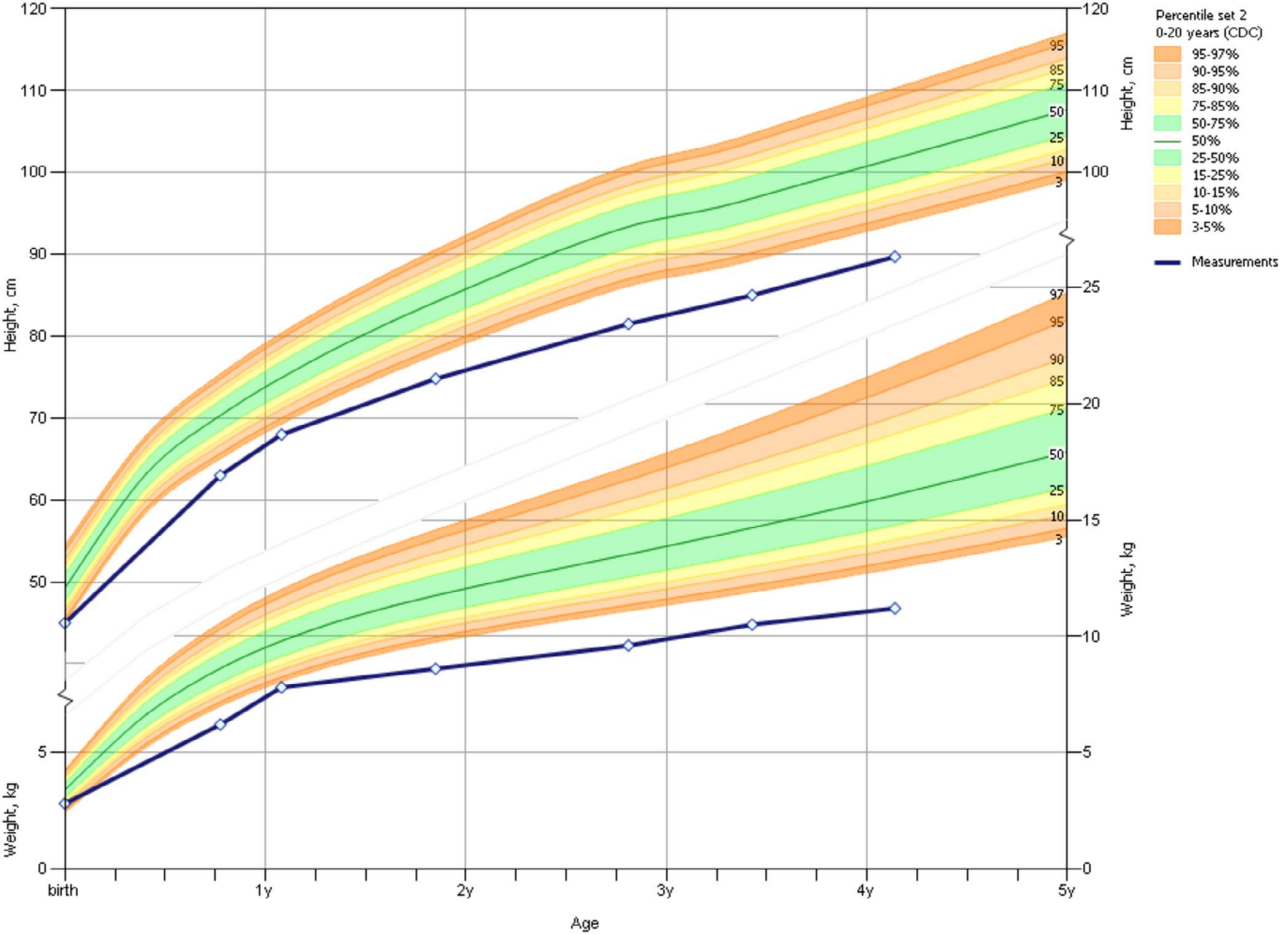
Growth Curves of Height, Weight and Age in the proband with short stature and normal girls. The blue curve shows the height/weight and age growth curve of each percentile of proband at different ages, and the other color curves show the height/weight and age growth curve of each percentile of normal children of 0‐4 years old girls.

The proband who initiated the visit to the endocrinology and genetics metabolism clinic was a 4‐year‐old girl who came with a concern of “slow growth”. She was delivered spontaneously at 39‐week gestation, had a birth weight of 2.8 kg, without feeding issues, and was the first child of the Chinese parents. Neonatal clinical examination at the local hospital did not reveal any abnormalities. At 8 months old, length was found to be located in P3 of the children's growth and development curve during a routine health examination, and dysplasia of the middle part of the face was noted. The child has experienced delayed growth for nearly 4 years (Figure 1). The height and weight of the child were normal at birth and gradually decreased in the first year after birth. With the increase of age, the difference in height and weight between the child and the normal girls gradually widened. At the age of 4, the average height of the child gradually began to be lower than 3.75 SD. The patient has a normal diet, exercises regularly, and has regular sleep patterns. There are no sensory impairments, abdominal pain, diarrhea, frequent urination, or increased thirst. She has received routine vaccinations, has no previous history of recurrent infections, surgical injuries, or allergies. Family members deny any consanguineous marriage or history of genetic disorders.

## 3 Differential Diagnosis, Investigations and Treatment

The patient's physical examination revealed nothing unremarkable, except for a skeletal abnormality known as “pectus excavatum” The potential etiology was extensively examined. Normal chromosomal karyotype, uterine adnexal ultrasound, and sex hormone levels ruled out Turner syndrome. She did not exhibit malnutrition, apart from malnutrition‐induced short stature. Furthermore, her thyroid function, adrenocorticotropic hormone, cortisol, and sex hormone levels were within normal ranges, as was her peak growth hormone level, ruling out growth hormone deficiency and multiple pituitary hormone deficiencies. Her liver and kidney function, glucose and lipid metabolism, as well as other test results, were all normal, thus eliminating the possibility of chronic systemic diseases. Normal results from pituitary MRI and tumor marker tests also ruled out tumor infiltration and brain damage. Pituitary MRI and a full‐length lateral spine X‐ray examination revealed no significant abnormalities. Limb X‐ray examination showed normal results without any signs of Madelung deformity. According to bone age assessment, the patient's bone age is 4 years.

**FIGURE 2:**
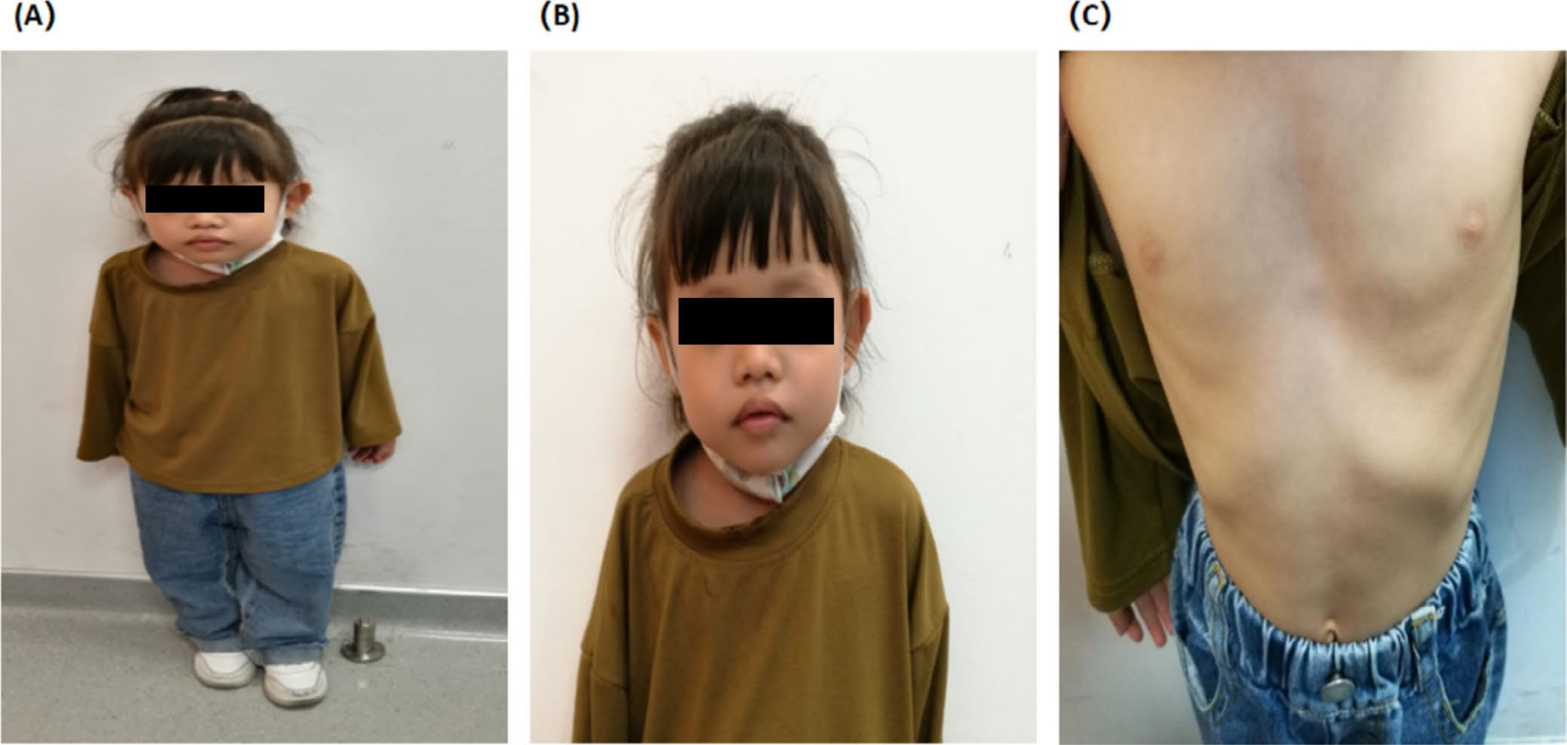
Photograph showing the height and the facial characteristics of the 4 years and 2 months old girl presented in this case study. (A) Her height is 3.75 SD lower than that of normal healthy children of the same race, age and sex. (B) She presented with midface retraction, a long face with lip protrusion, frontal bossing, sparse eyebrows, high palate, ears slightly forward. (C) Her sternum is funnel‐shaped, is a congenital malformation.

The patient's height is 89.7 cm (−3.75 SD), weight is11.2 kg, body mass index is 13.5 kg/m^2^, size emaciation, whose unique facial and physical features are shown in (Figure 2). She has slightly sparse eyebrows, lip protrusion, frontal bossing, high palate, ears slightly forward, long eyelashes, a square tip to her nose, normal columella, prominent two front teeth, normal tooth number, and upper lip is slightly upturned, funnel chest. There are no coffee spots on her skin; no abnormalities in the heart and lungs. The liver and spleen cannot be touched below the ribs. There was no hyperactivity or decrease in muscle tone of the extremities. Assessment of child sexual development is in Tanner stage I. The patient has normal motor, hearing, vision, and intellectual development. However, the patient's language development was delayed. The preliminary clinical diagnosis of the patient was short stature and developmental delay, and the genetic etiology was further clarified by the trio‐WES.

## 4 Results

**FIGURE 3:**
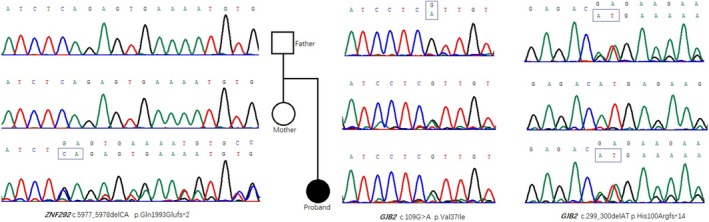
Pediatric family genealogy and Sanger sequence map. Sanger sequencing confirms de novo c.5977_5978del (p.Gln1993fs) in the ZNF292 gene in the patient; Maternal c.109G > A (p.Val37Ile) heterozygous variant and paternal c.299_300del (p.His 100 fs) heterozygous variant n theZNF292 gene in the patient.

The WES revealed the frame‐shift variant c.5977_5978del (p.Gln1993Glufs*2) in *ZNF292*. The candidate mutations were verified by Sanger sequencing. The findings from Sanger sequencing validated the existence of the ZNF292 gene variant in the patient while concluding that the parents possessed the normal, suggesting that the variant arose spontaneously. According to ACMG mutation classification standard, they can be classified as “likely Pathogenic” (PVS1 + PM2_Supporting), the specific evidence is as follows: the mutation is frameshift mutation, which belongs to functional deletion mutation, and it is predicted that it may lead to the early termination of ZNF292 gene translation (PVS1); As of April 2024, dbSNP, gnomAD, ClinVar and local databases have not been included (PM2_Supporting). In addition, it should be noted that *GJB2* gene c.299_300del and c.109G>A mutations heterozygous variants inherited from her father and mother, respectively (Figure 3).

ZNF292, located at 6q14.3 (chr6:87151803–87265943), contains 8 exons. The final exon is the largest, and the gene is responsible for coding all 16 highly conserved zinc fingers found in the 2723‐amino‐acid‐long predicted protein. Zinc finger domain‐containing proteins are essential for maintaining genome integrity, stability, and regulating transcription. Specifically, three of these zinc fingers (10–12) are involved in binding DNA at the growth hormone promoter. In somatotrophs, they work in conjunction with the α‐helix of the finger, aided by the conserved linkers between the fingers, to activate transcription [12]. ZNF292 is a zinc finger transcription factor that is dependent on growth hormone and falls under the kruepel C2H2‐type zinc finger protein family. Numerous research studies have identified ZNF292 as a gene involved in suppressing tumors, characterized by its significantly higher expression in normal tissues adjacent to tumors compared to its lower expression levels within the tumor tissues [13, 14]. Knockdown of ZNF292 led to increased rates of cell proliferation, enhanced colony formation capabilities, and progression through the cell cycle [15]. Mechanically, the depletion of ZNF292 triggered the upregulation of downstream genes at the transcriptional level, ultimately facilitating the progression of the cell cycle. Furthermore, mutations in the ZNF292 gene have been found in liver, colon, gastric, and bone marrow cancers. These mutations may play a role in tumorigenesis by modifying the functions of tumor suppressor genes within the cancer cells [16, 17]. The ZNF292 gene has been identified as a tumor suppressor that plays a crucial role in the development and advancement of tumors.

## 5 Discussion

**FIGURE 4:**
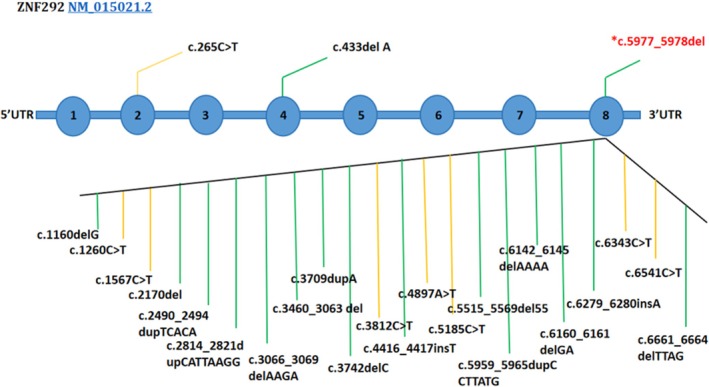
The distribution of 24 reported variants in ZNF292 shows that truncating mutations (frameshift, nonsense) are mostly found in exon 8, the largest and most terminal exon of the gene. Variants of the ZNF292 gene in the main cohort are categorized by type, with nonsense variants represented in yellow and frameshift variants in green, this case report demonstrates a de novo frameshift mutation highlighted in red.

ZNF292 is abundantly present in the developing human brain, with particularly high levels in the cerebellum. Its peak expression occurs during the prenatal stage, contributing significantly to brain function and neurodevelopment. Variants inherited de novo or in a dominant manner in ZNF292 have been linked to various neurodevelopmental characteristics such as ID and ASD [18]. In 2014, first reported [9] a de novo heterozygous c.265C‐T transition (NM_015021.2) in exon 2 of the ZNF292 gene, resulting in an arg89‐to‐ter (R89X) substitution. Subsequently, at a 17‐year‐old male of northern European occurrence of the mutation that extraction with mildly impaired intellectual development, speech and gross motor delays, attention deficit‐hyperactivity disorder, and visuospatial dyspraxia. Until now, Mirzaa et al. [18] reported that corroborated a total of 24 heterozygous nonsense or frameshift mutations in the ZNF292 gene, which occurred primarily in the last exon (Figure 4). The mutations occurred as de novo events, 2 of these mutations were recurrent. Only one of the mutations was inherited from the mother; the exception was present only once. ZNF292‐related disorders included developmental delays and ID. Additional neurodevelopmental features comprised speech delays, ASD, attention‐deficit hyperactivity disorder, tone abnormalities, brain MRI abnormalities, and epilepsy. Non‐neurological features encompassed growth failure (weight/height < 2SD), subtle facial dysmorphism, microcephaly (OFC < 2SD), ocular abnormalities, feeding issues, constipation, and cardiac abnormalities.

Moreover, Table 1 illustrates that over 13 pathogenic or likely pathogenic mutations have been documented in the ZNF292 gene within the DECIPHER database. Nonetheless, some of the phenotypes observed in our case patient were either not previously recorded or exhibited variations. For example, delayed speech and language development and short stature have also been reported in other previous reports [19, 20, 21], which is in accordance with our observations. It is worth noting that speech delay is prominent in our case patient; she walked by 17 months of age and vocalized “mama, baba” simple vocabulary at 4 years old. Maybe her parents did not notice that she had regression of speech and language development early, but she speaks normally at present. The mechanism by which ZNF292 variants disrupt human brain development and behavior is ambiguous. The two most likely possibilities for LOF to become the basis of disease are evasion of nonsense‐mediated decay (NMD), leading to the expression of truncated proteins with gain‐of‐function or dominant negative effects, or simple haploinsufficiency [22]. Although IDs and attention deficit hyperactivity disorder were reported in ZNF292 as mostly clinical phenotypes, the patient in our case has not exhibited clinical manifestations with neurological symptoms yet. There are still residual risks of isolated behavioral issues in the future. Similarly, in our case, growth abnormalities were also observed including the child having short stature of 3.75 SD below the mean at age 4 years, with no hypotonia and hypertonia. She had subtle facial dysmorphism, most notably protrusion of the forehead, upturned lips, nasal root collapse, and ears slightly forward. To date, the observed malformation features have been non‐specific, leading to potentially lower clinical recognition in individuals with pathogenic ZNF292 variants. Notably, the pectus excavatum of skeletal abnormalities has not been reported in the originally reported cases.

**TABLE 1:** Variants identified in the ZNF292 gene in 13 patients.

Patient	Sex	Transcript/location (GRCh38)	ZNF292 variants	Effect on protein	Type of variant	Pathogenicity	Inheritance/genotype	Phenotypes
266507	46XX	6:87259893‐87259897	c.6265_6268del	p.Lys2089Arg	Frameshift	Likely pathogenic	Unknown/heterozygous	Abnormality of the outer ear; Global developmental delay
269101	46XY	6:87255802	c.2173C>T	p.GIn725Ter	Stop_gained	Likely pathogenic	De novo/heterozygous	Delayed speech and language development; Inguinal hernia; Intellectual disability; Oromotor apraxia
277962	46XX	6:87255802	c.2173C>T	p.Arg358Cys	Missense	Uncertain	De novo/heterozygous	Constipation; Delayed CNS myelination; Drooling; Microcephaly; Plagiocephaly; Severe global developmental delay; Visual impairment
283609	46XY	6:87259787‐87259791	c.6159_6162del	p.GIu2054Val	Frameshift	Likely pathogenic	De novo/heterozygous	2–3 toe syndactyly; Deeply set eye; Delayed speech and language development; Microcephaly; Moderate global developmental delay; Narrow chest; Short columella; Short stature; Sparse scalp hair; Thin vermilion border; Wide nose
285110	46XY	6:87259787‐87259789	c.61596160del	p.Glu2054Lys	Frameshift	Pathogenic	De novo/heterozygous	Abnormality of skin morphology; Intellectual disability, moderate; Microcephaly; Multiple cafe‐au‐lait spots; Neuroblastoma
306361	46XX	6:87260452	c.6823C>T	p.Arg2275Ter	Stop_gained	Uncertain	De novo/heterozygous	Atrial septal defect; Clinodactyly of the 5th finger; Delayed speech and language development; Epicanthus; Intrauterine growth retardation; Motor delay; Short stature
412265	46XX	6:87258072‐87258076	c.4444_4447del	p.Ser1483Lys	Frameshift	Uncertain	De novo/heterozygous	Abnormal cerebellum morphology; Dysarthria; Hand tremor; Intellectual disability, mild
421793	46XY	6:87259845‐87259849	c.6217_6220del	p.Asn2074His	Frameshift	Likely pathogenic	Maternally inherited/heterozygous	Attention deficit hyperactivity disorder; Autistic behavior; Global developmental delay
422644	46XX	6:87259162	c.5533C>T	p.GIn1845Ter	Stop_gained	Pathogenic	Maternally inherited/heterozygous	Abnormal facial shape; Attention deficit hyperactivity disorder; Delayed speech and language development
454294	46XY	6:87255388‐87255390	c.1760_1761del	p.His587Arg	Frameshift	Likely pathogenic	De novo/heterozygous	Intellectual disability, mild; Nystagmus; Seizure
454349	46XY	6:87257575‐87257576	c.3947del	p.Asn1317Met	Frameshift	Pathogenic	De novo/heterozygous	Abnormal aggressive, impulsive or violent behavior; Autism; Developmental regression; Hyperactivity; Intellectual disability; Postnatalmacrocephaly; Self‐injurious behavior; Short corpus callosum; Socially inappropriate behavior
517120	46XY	6:87257293	c.3664G>T	p.Glu1222Ter	Stop_gained	Likely pathogenic	Maternally inherited/heterozygous	Delayed speech and language development; Intellectual disability
521470	46XX	6:87254891	c.1262A>G	p.Asn421Ser	Missense	Uncertain	De novo/heterozygous	Cerebral visual impairment; Dysphagia; Global developmental delay; Primary microcephaly; Seizure

The ZNF292 gene displays various mutation types, including missense, splice site, nonsense, and frameshift mutations, with missense mutations being the most prevalent. These identified variants in ZNF292 are associated with a loss‐of‐function impact. However, our comprehension of ZNF292 defects leading to clinical symptoms remains limited. This study presents the case of a 4‐year‐old girl from a Chinese family exhibiting short stature, developmental delay, pectus excavatum, and nonspecific facial features. She carries a newly identified frameshift variant in ZNF292, consistent with previous findings, thereby broadening the clinical phenotype and expanding the mutation spectrum associated with ZNF292.

The evaluation of short stature with developmental delay should integrate auxological, developmental endocrine, genetic, nutritional, and systemic factors to differentiate endocrine defects (e.g., GHD), genetic syndromes (e.g., Noonansyndrome) or systemic conditions (e.g., celiac disease). The association between ZNF292 mutations and short stature with developmental delay adds a new layer to the heterogeneous etiology of growth disorders. While traditional diagnostic algorithms prioritize endocrine testing (e.g., GH‐IGF1 axis, thyroid function) and chromosomal analysis (e.g., Turner syndrome), our data suggest that ZNF292 defects represent a non‐syndromic genetic form with unique molecular signatures. The phenotypic overlap between ZNF292‐related disorder and other short stature syndromes underscores the importance of molecular testing in differential diagnosis. Unlike Noonan syndrome (PTPN11 mutations), ZNF292 cases rarely exhibit facial dysmorphism or congenital heart disease, while their normal IGF‐1 levels distinguish them from GH deficiency [23]. Notably, the co‐occurrence of developmental delay and absence of syndromic features should prompt ZNF292 screening, particularly in exome‐negative idiopathic cases.

Meanwhile, ZNF292 is also a tumor suppressor gene (TSG), which may play an important role in colorectal and liver cancers. Its predicted role in genomic stability (via conserved zinc‐finger domains) warrants caution. Analogous to BRCA1, loss of ZNF292 function might impair DNA damage repair, theoretically increasing susceptibility to solid tumors or leukemias [24]. We propose tiered surveillance: annual physical exams for all patients, supplemented by biennial abdominal ultrasound and serum AFP in those with truncating mutations or family history of early‐onset cancers.

The use of rhGH in ZNF292‐mutated individuals remains controversial. While GH may improve height velocity in non‐GHD short stature (e.g., Turner syndrome), its efficacy here is unproven, and theoretical risks outweigh potential benefits in high‐risk subgroups. Specifically, patients with truncating mutations or prior neoplasms should avoid rhGH due to concerns about synergistic oncogenic effects, whereas those with mild missense variants may trial therapy under close monitoring for scoliosis and glucose intolerance [25].

In addition, in our reported case, the c.299_300del (p.His100fs) and c.109G > A (p.Val37Ile) compound heterozygous mutations in the GJB2 gene that were respectively inherited from her parents were detected in the girl. The patient had normal hearing. The genetic pattern of GJB2 is mainly autosomal recessive inheritance, among which c.235delC, c.299‐300delAT, and c.109G > A are common hot spot mutations in Chinese deafness [21]. Among the mutations of the GJB2 gene, there are generally two types of mutations. Truncated mutations (such as c.299‐300delAT) generally cannot form protein structures, so they cause severe hearing loss; however, non‐truncated variants (such as c. 109G > A) usually only affect single or multiple amino acids and can express relatively complete protein structures. As far as we know, c.299_300delAT (p.His100fs) accounts for about 4.3% of the hearing‐impaired population, and the carrier rate is about 0.4% in the normal people [26]. Among them, the heterozygous rate of GJB2 c.109G > A (p.Val37Ile) mutation in the Chinese Han population is as high as 12%, and the homozygous rate is close to 0.4% [27]. However, this mutation has significant incomplete dominance, and a large number of homozygous mutation or compound heterozygous mutation carriers have normal hearing. A series of recent studies [28, 29, 30] have shown that the homozygous mutation and compound heterozygous mutation of c.109G > A (p.Val37Ile) detected in mild to moderate deafness and delayed deafness populations are higher than those in the normal hearing control population, which are typical genetic susceptibility mutations. In the Chinese Han population, only 14.6% of GJB2 gene c.109G > A (p.Val37Ile) biallelic mutations manifest congenital deafness, which can cause 0.4 dB hearing loss per year [31]. The c.109G > A mutation combined with the c.299‐300delAT heterozygous mutation in the GJB2 gene has been reported to possibly cause moderate to severe sensorineural hearing loss [32, 33, 34]. The hearing phenotype caused by the c.109G > A (p.Val37Ile) compound heterozygous mutation is quite different in different individuals. It is recommended that follow‐up hearing examinations be performed to accurately evaluate hearing levels, and regular hearing monitoring can be performed. Hence, avoid being dumb due to deafness.

In conclusion, the present study reported on a Chinese patient with a novel frameshift mutation in ZNF292gene (c.5977_5978del). The patient was diagnosed with pectus excavatum, short stature, and language developmental delay, which had not been properly identified before. Various analyses of WES data indicated that no other pathogenic gene was involved in the phenotype. This research broadens the range of mutations in the ZNF292 gene and deepens our understanding of this phenotype. As far as we know, this is the first case report of a novel frameshift function mutation of the ZNF292 gene in Asia. Continuous monitoring and timely intervention for possible progressive hearing impairment is recommended, as it can produce negative effects such as behavioral problems and poor learning abilities in children's speech and language development. Integrating ZNF292 into diagnostic workflows refines the classification of syndromic short stature, while its putative tumor‐suppressor function calls for long‐term oncologic vigilance. Future studies should establish genotype–phenotype correlations, clarify malignancy risks through registries, and explore targeted therapies beyond GH.

## Author Contributions

**Li Dongxue:** formal analysis, funding acquisition, writing – original draft. **Yao Ruen:** data curation, writing – review and editing. **Yu Ying:** conceptualization, investigation. **Wang Jie:** project administration, resources. **Chen Liting:** data curation, methodology. **Li Wei:** data curation, investigation. **Lu Chunjiao:** data curation, resources. **Chen Rikun:** investigation, resources. **Li Cuiyun:** data curation, writing – review and editing.

## Ethics Statement

The studies involving human participants were reviewed and approved by Sanya Women and Children's Hospital Managed by Shanghai Children's Medical Center (SYFYIRB2023002).

## Consent

Written informed consent to participate in this study was provided by the participants' legal guardian/next of kin.

## Conflicts of Interest

The authors declare no conflicts of interest.

## Data Availability

The sequencing raw data are submmited to the GSA for human. It's numbered subHRA011104 (https://ngdc.cncb.ac.cn/gsa‐human/s/j7W7qnoF). They are available from the corresponding author on reasonable request.
